# Pistachio consumption modulates DNA oxidation and genes related to telomere maintenance: a crossover randomized clinical trial

**DOI:** 10.1093/ajcn/nqz048

**Published:** 2019-05-03

**Authors:** Silvia Canudas, Pablo Hernández-Alonso, Serena Galié, Jananee Muralidharan, Lydia Morell-Azanza, Guillermo Zalba, Jesús García-Gavilán, Amelia Martí, Jordi Salas-Salvadó, Mònica Bulló

**Affiliations:** 1 Human Nutrition Unit, Faculty of Medicine and Health Sciences, Institut d'Investigació Sanitària Pere Virgili, Rovira i Virgili University, Reus, Spain; 2 CIBER Fisiopatología de la Obesidad y Nutrición (CIBEROBN), Instituto de Salud Carlos III, Madrid, Spain; 3 Department of Nutrition, Food Sciences and Physiology; 4 Department of Biochemistry and Genetics, University of Navarra, Pamplona, Spain; 5 IdiSNA, Instituto de Investigación Sanitaria de Navarra, Pamplona, Spain

## Abstract

**Background:**

Telomere attrition may play an important role in the pathogenesis and severity of type 2 diabetes (T2D), increasing the probability of β cell senescence and leading to reduced cell mass and decreased insulin secretion. Nutrition and lifestyle are known factors modulating the aging process and insulin resistance/secretion, determining the risk of T2D.

**Objectives:**

The aim of this study was to evaluate the effects of pistachio intake on telomere length and other cellular aging-related parameters of glucose and insulin metabolism.

**Methods:**

Forty-nine prediabetic subjects were included in a randomized crossover clinical trial. Subjects consumed a pistachio-supplemented diet (PD, 50 E% [energy percentage] carbohydrates and 33 E% fat, including 57 g pistachios/d) and an isocaloric control diet (CD, 55 E% carbohydrates and 30 E% fat) for 4 mo each, separated by a 2-wk washout period. DNA oxidation was evaluated by DNA damage (via 8-hydroxydeoxyguanosine). Leucocyte telomere length and gene expression related to either oxidation, telomere maintenance or glucose, and insulin metabolism were analyzed by multiplexed quantitative reverse transcriptase-polymerase chain reaction after the dietary intervention.

**Results:**

Compared with the CD, the PD reduced oxidative damage to DNA (mean: −3.5%; 95% CI: −8.07%, 1.05%; *P* = 0.009). Gene expression of 2 telomere-related genes (*TERT* and *WRAP53*) was significantly upregulated (164% and 53%) after the PD compared with the CD (*P* = 0.043 and *P* = 0.001, respectively). Interestingly, changes in *TERT* expression were negatively correlated to changes in fasting plasma glucose concentrations and in the homeostatic model assessment of insulin resistance.

**Conclusions:**

Chronic pistachio consumption reduces oxidative damage to DNA and increases the gene expression of some telomere-associated genes. Lessening oxidative damage to DNA and telomerase expression through diet may represent an intriguing way to promote healthspan in humans, reversing certain deleterious metabolic consequences of prediabetes. This study was registered at clinicaltrials.gov as NCT01441921.

## Introduction

Telomere attrition is a natural phenomenon widely recognized as one of the hallmarks of aging. A large number of population-based studies have observed a decrease in leukocyte telomere length (LTL) in parallel with increased age (1). However, over the last decade a growing body of evidence has indicated that short telomeres are a relevant modifier of type 2 diabetes (T2D) risk and may be essential biomarkers that identify individuals at high future risk of T2D in clinical settings (2). Despite the fact that the mechanism(s) involved are not clear (3), several pieces of evidence support the idea that chronic systemic inflammation aggravates reactive oxygen species (ROS)–mediated telomere dysfunction, decreasing regenerative potential in multiple tissues and accelerating cellular aging in the absence of any other genetic or environmental factors (4).

Telomeres are specialized structures at the ends of chromosomes (i.e., TTAGGG repeats) that play a fundamental role in chromosome stability and integrity (5). As telomeres become shorter with each cell division, they activate a DNA damage response that leads to replicative senescence and anticipates the onset of age-associated diseases (6). In fact telomere length (TL) is linked to—and likely regulated by—exposure to proinflammatory cytokines and oxidative stress, with an effective autocrine and paracrine signaling activity that may contribute to insulin resistance (IR) (7). The enzyme responsible for the maintenance of TL is telomerase, a reverse transcriptase with catalytic activity (TERT). Telomerase helps to protect against both this telomere loss caused by chronic oxidative stress and cellular aging (8) by making additional copies of the TTAGGG repeats at the chromosome ends (9). Transcriptional regulation of *TERT* is tightly controlled, and evidence links the association of telomerase activity to *TERT*expression (10). Scientific findings on *TERT* regulation by microRNAs (miRNAs) have recently updated thinking regarding the biology of telomeres and telomerase (11).

Nutrition, oxidative damage, telomere shortening, and cell senescence represent a sequence of processes that play an important role in in vivo aging and longevity (12–14), with TL being the causal pathway between lifestyle (including diet) and risk of disease (15, 16). The association between diet and the shortening of telomeres is currently under examination. A number of studies have reported both a decrease and an increase in TL as a result of diet (17, 18). Nuts are a food matrix that is rich in vitamins, polyphenols and phytosterols with acknowledged antioxidant and anti-inflammatory properties, and are positively associated with TL (19).

Here, we took advantage of the EPIRDEM (Effect of Pistachio Intake on Insulin Resistance and Type 2 Diabetes Mellitus) study, a randomized, controlled, crossover nutrition intervention trial conducted in prediabetic subjects that demonstrated the beneficial effect of pistachio intake on glucose metabolism, IR, and systemic inflammation (20). We now aim to evaluate the effect of pistachio consumption on cellular aging parameters related to glucose metabolism and IR as a possible way of reversing some of the deleterious metabolic consequences of prediabetes.

## Research Design and Methods

### Study characteristics

The EPIRDEM study is a randomized, controlled, crossover trial comprising two 4-mo dietary intervention periods separated by a 2-wk washout interval, carried out in prediabetic subjects with the aim of evaluating the effect of pistachio intake on glucose metabolism (20). The primary outcome variables were to analyze glucose metabolism, IR, and inflammatory and oxidative risk markers. Changes in gene expression related to glucose metabolism and telomere maintenance were included as secondary outcomes. The trial was registered at clinicaltrial.gov as NCT01441921 and the institutional ethics committee approved the study protocol in September 2011. Written informed consent was obtained from all study subjects, and the protocols and procedures were conducted in accordance with the ethical standards of the Declaration of Helsinki.

### Study population

Eligible participants were men and women aged between 25 and 65 y, with BMI ≤35 kg/m^2^ and fasting plasma glucose concentrations between 100 and 125 mg/dL (prediabetes condition) following the protocol (20). Subjects were excluded if any of the following criteria applied: *1*) T2D or use of oral antidiabetic drugs; 2) alcohol, tobacco, or drug abuse; *3*) frequent consumption of nuts or known history of nut allergy; *4*) use of plant sterols, psyllium, fish oil supplements, multivitamins, vitamin E, or other antioxidant supplements; *5*) bad dentures, making it difficult to chew pistachios; *6*) following a vegetarian or hypocaloric diet to lose weight; *7*) being pregnant or hoping to become pregnant 9 mo before or during the study or lactating 6 wk before or during the study; *8*) significant liver, kidney, thyroid or other endocrine diseases; or *9*) medical, dietary, or social conditions that may hinder compliance with the intervention. Participants were recruited from primary care centers in Reus, Spain.

### Study design

The study was designed as a crossover trial with a 15-d run-in period preceding the 4-mo dietary treatment period in each arm. In the 15-d run-in period participants were advised to follow a normocaloric diet (50% energy [E%] as carbohydrates, 15 E% as protein and 35 E% as total fat). After this period, subjects were randomly assigned to 1 of the 2 series with the use of a computer-generated random table: an initial control diet (CD) followed by the pistachio-supplemented diet (PD) or an initial PD followed by the CD. Participants assigned to the PD were supplemented with 2 oz pistachios/d (57 g/d, half roasted/half roasted and salted). The CD was free of nuts, and the energy intake from other fatty foods, mostly olive oil, was adjusted to compensate for the energy from the pistachios included in the PD.

### Anthropometry, body composition and blood pressure

Weight and waist circumference were determined at baseline after the 15-d run-in and at the beginning and end of each intervention arm, with subjects wearing light clothes and no shoes. BMI was calculated at the beginning and end of each 4-mo intervention period. Body composition was measured by bioelectrical impedance analysis (Human-Im-Scan; Dietosystem). Blood pressure was measured in the nondominant arm with a validated semiautomatic oscillometer (HEM-705CP; OMRON) in duplicate with a 5-min interval between each measurement.

### Biological samples, biochemical and molecular parameters

Blood samples were obtained after a 12-h fast at baseline and at the end of each intervention period. Plasma fasting glucose was measured by standard enzymatic assays. Insulin was determined with the use of a MILLIPLEX MAP Plex Kit (Merck Millipore). Insulin resistance was estimated according to the HOMA-IR.

### Assessment of DNA damage

8-Hydroxydeoxyguanosine (8-OHdG) concentrations were measured in plasma samples at baseline and after each intervention period. The stability of plasma 8-OHdG measurement provides a sensitive and noninvasive way to evaluate the extent of cellular oxidative stress and DNA damage. The assay was carried out with 20 µL of plasma sample and quantitative estimation of 8-OHdG was performed with the use of an OxiSelect Oxidative DNA damage ELISA Kit in accordance with the manufacturer's instructions (Cell Biolabs, Inc.). The colorimetric substrate was monitored at a wavelength of 450 nm on an ELISA conventional plate reader (Fluoroskan Ascent; Thermo Fisher Scientific).

### Telomere length measurement

TL was measured with the use of a monochrome multiplex real-time quantitative PCR method described elsewhere (21). Briefly, this method performs in a single reaction the quantification of the relative copy numbers of telomeres and a single copy gene, and TL is expressed as a ratio of these 2 parameters. DNA was extracted with the use of a DNA blood extraction kit (Pure Link Genomic DNA, Invitrogen). A calibration curve with a reference DNA sample (150–2.34 ng/µL in 2-fold dilutions) was included in each 384-well plate and used for the relative quantification. The master mix used contained a QuantiTect Syber Green PCR kit (Qiagen), telomere primer pairs, albumin primer pairs, and ultrapure water to complete the final volume. The primer pair telg and telc (final concentration 900 nM each) were combined with the single-copy gene *albu* and *albd* (final concentration 900 nM each). The primer sequences were telg (5′-ACACTAAGGTTTGGGTTTGGGTTTGGGTTTGGGTTAGTGT-3′), telc (5′TGTTAGGTATCCCTATCCCTATCCCTATCCCTATCCCTAACA-3′), albu (5′- CGGCGGCGGGCGGCGCGGGCTGGGCGGAAATGCTGCACAGAATCCTTG-3′) and albd (5′-GCCCGGCCCGCCGCGCCCGTCCCGCCGGAAAAGCATGGTCGCCTGTT-3′). All primers were from Sigma Aldrich and were purified by the manufacturer through the use of HPLC.

Experiments were conducted in a 384-well plate and all samples were run in triplicate. We carried out the following multiplex real-time quantitative PCR protocol in a CFX384 Touch Real-Time PCR system (BioRad): 15 min at 95°C for enzyme activation followed by 2 cycles at 95°C for 15 s and 49°C for 15 s, and 35 cycles of 15 s at 95°C, 10 s at 63°C, 15 s at 74°C (first signal acquisition), and 15 s at 88°C (second signal acquisition). For each sample, we generated a melting curve from 45°C to 95°C, ramped at 0.2°C/s.

### qRT-PCR

Total mRNA isolation from whole-blood samples was performed with the use of a Tempus Spin RNA Isolation Kit (Ambion) in accordance with the manufacturer's instructions. Final RNA concentration and purity were measured with a Qubit 4 Fluorometer (Invitrogen). A 1-μg portion of total mRNA per sample was reverse-transcribed with a High Capacity cDNA Reverse Transcription Kit (Invitrogen) in accordance with the manufacturer's instructions. Incubation was at 25°C for 10 min, reverse transcription was at 37°C for 120 min, and inactivation was at 85°C for 5 min. cDNA containing 180 ng RNA/sample was isolated from blood lymphocytes of CD or PD participants in all time periods.

Total RNA (including miRNA) was isolated from plasma samples with the use of a mirVana PARIS Isolation Kit (Applied Biosystems) in accordance with the manufacturer's protocol as described elsewhere (22). We selected 7 human circulating miRNAs (hsa-miR-15a-5p, hsa-miR-21-5p, hsa-miR-29b-3p, hsa-miR-126-3p, hsa-miR-192-5p, hsa-miR-223-3p, and hsa-miR-375) widely related to glucose metabolism, IR status, pre-diabetic status and biomarkers of T2D, based on the use of updated reviews and databases (22).

### Screening step: gene expression array

In the initial screening step we profiled gene expression in a randomly selected representative cohort of 10 subjects (analyzed at baseline and after the intervention period, i.e., pistachio–control or control–pistachio). A total of 94 genes (**Supplemental Table 1**) were quantified with the use of Custom TaqMan Array Cards (Applied Biosystems) preconfigured in a 96-well format and spotted on a microfluidic card (2 replicates/assay). The genes included were a selection of telomere maintenance, DNA damage, oxidative stress, and diabetes-related genes based on the assessment of updated reviews and databases. The real-time RT-PCR amplifications were then run on a 7900HT Fast Real-Time PCR System Sequence Detection System (Applied Biosystems).

Data from qPCR were obtained by SDS version 2.2 (Life Technologies, version 2.4) and processed by RQ Manager version 1.2 software: the relative expression was calculated through the use of the comparative ΔCt method. A threshold cycle (Ct) >45 was considered the threshold for nonexpressed genes. The relative quantification (RQ) of gene expression was determined with the use of the comparative ΔΔCt: RQ = 2^−ΔΔCt^ with ΔCt = Ct (target gene) – Ct (endogenous gene) and ΔΔCt = ΔCt (PD or CD) – ΔCt (CD or PD). Gene expression was considered upregulated if there was a 1.5-fold change in the levels within the PD diet and CD diet. Thermal cycling conditions were as follows: 50°C for 2 min, 92°C for 10 min followed by 45 cycles at 97°C for 1 s and 62°C for 20 s. The assay ID for the genes is shown in Supplemental Table 1. *HPRT1* and *18S* were both used as reference genes for target gene normalization. In total, 3000 ng of cDNA was mixed with TaqMan Fast Advanced Master Mix (Applied Biosystems).

### Validation step: gene expression by qRT-PCR

To confirm and validate the gene expression signature panel we next established a custom TaqMan low-density array set and validated across all 49 participants to identify putative candidate genes. Genes were considered differentially modulated by treatments based on gene expression levels (Cq values <45 in PD-CD or CD-PD). We also carried out a bibliographic search to select genes that had previously been linked to telomere maintenance, oxidation, and glucose metabolism. In the end, 22 of the genes (*ADRB3, BLM, CHEK2, FOXP3, GPX1, GPX2, INS, ISG15, MTFP1, NCL, NEROD1, NOX5, PPP2R1A, PRDX1, RAD1, RTEL1, SIRT2, SIRT6, SSB, TERT, TINF2*, and *WRAP53*) were chosen as candidates for further confirmation and validation across all participants by qRT-PCR (Supplemental Table 1).

All measurements were performed in duplicate and qPCR data were acquired with the use of sequence detector software (SDS version 2.4; Applied Biosystems). The expression of the genes analyzed was normalized by the mean of *GAPDH* and *HPRT1* and the normalized expression was calculated for individual samples through the use of 2-ΔCq methods. The inclusion criteria for significantly upregulated genes, as reported previously (22), were: *1*) a mean 1.5-fold change; *2*) *P* < 0.05 for comparisons of both intervention treatments; and *3*) a Cq value ≤ 45. Changes in expression were shown as the ratio between final and baseline values. Of the initial 22 genes included in the validation, 5 (*ADRB3, INS, GPX2, NEROD1, NOX5*) were not further analyzed due a high proportion of missing values (≥40% of the samples).

### Statistical analysis

The descriptive data for the participants during the intervention periods are shown as means, with 95% CIs for continuous variables and numbers (%) for categoric variables. Normal distribution and homogeneity of the variances were evaluated with Levene's test and normalized relative log10 ratios were used when necessary. The antilog-transformed values are reported. Differences in all variables were evaluated by ANOVA, with the intervention diet as the independent and repeated-measures factor. Diet sequence (order of diet treatments, i.e., PD–CD, CD–PD) was analyzed as the independent factor for gene expression analysis, DNA oxidation, and TL. As it was significant in the case of TL, for this outcome only data from the first intervention period was considered. Paired *t* tests were performed to evaluate changes within each specific intervention period. The ratio of expression relative to the baseline of each mRNA is shown. Values >1 mean upregulation throughout the intervention period and values <1 mean downregulation. DNA oxidation is shown as a percentage change from the baseline, whereas TL is shown as age- and baseline-adjusted changes. Pearson's correlation coefficients were used to evaluate whether the changes (end/beginning of each intervention period) in plasma concentrations of different mRNAs correlated with the clinical parameters for glucose and insulin metabolism, together with DNA oxidation and TL (end/beginning) in the whole population, regardless of the intervention period. All the analyses were performed with R version 3.4.0 statistical software. A 2-sided *P* value of <0.05 was considered significant.

## Results

A total of 108 subjects were assessed for eligibility. After excluding those who declined to participate (*n* = 30) and those who did not meet the inclusion criteria (*n* = 24), 54 participants were randomly assigned to 1 of the 2 intervention sequences (i.e., PD–CD or CD–PD). Five participants dropped out for personal reasons and no nucleic acid samples were available (either at baseline or follow-up). Thus a total of 49 subjects successfully completed the study and are included in the analysis (**Supplemental Figure 1**). The baseline characteristics of these 49 study participants are shown in **Table 1**. No significant differences were observed between dietary interventions at baseline in any of the analyzed parameters. Similarly, baseline DNA oxidation and TL did not differ between dietary interventions (*P* = 0.458 and *P* = 0.452, respectively) (**Supplemental Table 2**).

**TABLE 1:** Baseline characteristics of the study population before the start of the study^1^

Variables	Subjects (*n* = 49)
Females, *n* (%)	23 (46.9)
Age, y	55.7 (53.9, 57.4)
Weight, kg	77.0 (74.2, 79.9)
BMI, kg/m^2^	28.9 (28.2, 29.6)
Waist circumference, cm	94.5 (92.6, 96.4)
Systolic blood pressure, mm Hg	134 (130, 138)
Diastolic blood pressure, mm Hg	81 (79, 83)
Total cholesterol, mg/dL	212.7 (204.0, 221.5)
LDL cholesterol, mg/dL	135.0 (126.4, 143.6)
HDL cholesterol, mg/dL	54.4 (50.4, 58.4)
Triglycerides, mg/dL	116.7 (102.8, 130.7)
Glucose, mg/dL	114.3 (109.9, 118.7)
Insulin, mU/mL	12.4 (10.8, 14.1)
HOMA-IR	3.59 (3.04, 4.14)
HbA1c, %	5.93 (5.81, 6.05)
Dyslipidemia, *n* (%)	26 (53.1)
Hypertension, *n* (%)	22 (44.9)

^1^Data are given as means (95% CIs) or numbers (%). HbA1c, glycated hemoglobin.

### Oxidative DNA damage after the intervention

**FIGURE 1:**
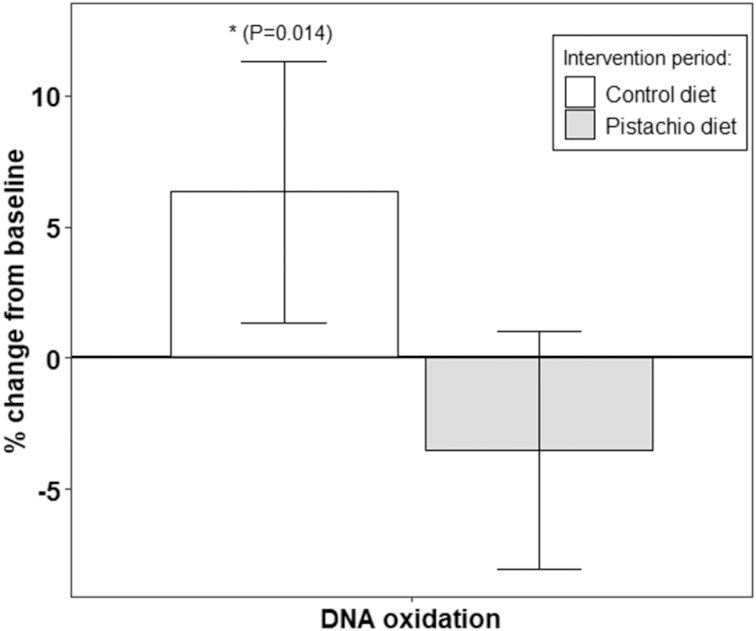
Percentage changes in plasma 8-OHdG levels in the course of the interventions. Results are means (95% CI) of 2 replicate samples for each point. *Significant compared with pistachio diet (*P* < 0.05). *n* = 49, both periods are considered. 8-OHdg, 8-hydroxydeoxyguanosine.

8-OHdG, a residue generated by the attack of ROS on DNA, was measured in plasma samples as an indicator of oxidative DNA damage. As shown in **Figure 1**, 8-OHdG concentrations significantly increased (mean: 6.34%; 95% CI: 1.36%, 11.32%; *P* = 0.014) during the CD period and showed a tendency to decrease (mean: −3.5%; 95% CI: −8.07%, 1.05%; *P* *=* 0.086) during the PD period. The differences in changes between intervention periods were significant (*P* = 0.009).

### Telomere length

**FIGURE 2:**
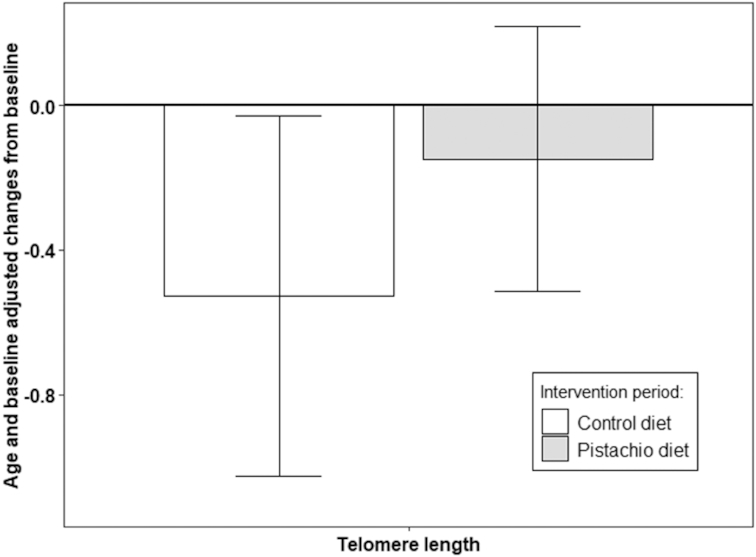
Multivariable-adjusted differences (95% CI) in changes in TL *z* score after the intervention period, adjusted by age and baseline TL. There was no significant difference between the changes. *n* = 49, limited to the first intervention period due to carryover effect. TL, telomere length.

No significant differences between changes in the LTL between intervention periods were reported (*P* = 0.237) (**Figure 2**). Interestingly, changes in TL were significantly and negatively correlated with changes in HOMA-IR (*r* = −0.203, *P* = 0.021).

### Gene expression modulation by the intervention diet

**FIGURE 3:**
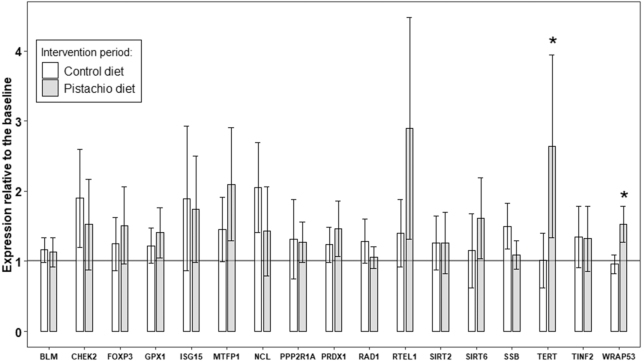
Expression relative to the baseline of the genes across intervention diets. Data are given as means (95% CI). Values equal to 1 mean the same expression at baseline and at the end of a particular period, whereas values >1 mean upregulation throughout the intervention period and <1 mean downregulation. *Significant differences in changes between dietary interventions (*P* < 0.05). *n* = 49, both periods are considered.

As indicated in **Figure 3**, 2 genes were differentially modulated by treatments (dietary interventions) based on gene expression levels (Cq values ≤45 in PD–CD). Validation results based on the qRT-PCR data show that genes related to TL maintenance (*TERT*, P < 0.043; and *WRAP53, P* < 0.0013) were significantly upregulated in the PD compared with in the CD. However, the expression of the remaining 15 genes did not significantly differ between the PD and CD intervention periods (**Supplemental Table 3**). In addition, we found a positive and significant correlation between changes in TL and changes in *TERT* expression (*r* = 0.128, *P* = 0.044).

### *TERT* expression and plasma glucose, insulin, and HOMA-IR

**FIGURE 4:**
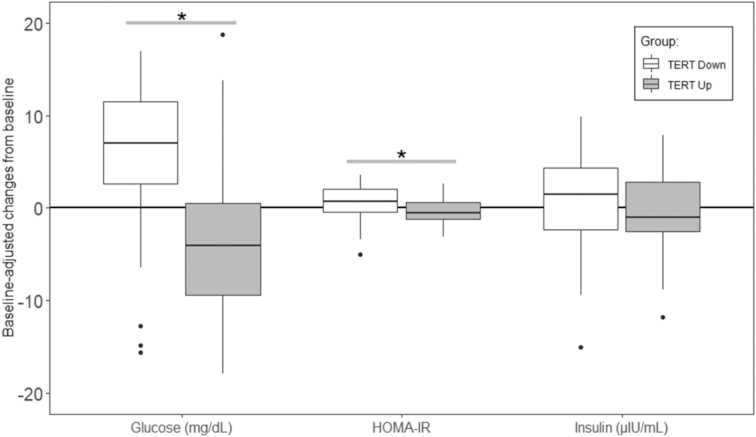
Boxplots of the associations between *TERT* regulation (i.e., upregulation or downregulation) and baseline-adjusted changes in biochemical parameters related to glucose metabolism, insulin resistance, and metabolic derangements associated with T2D. Gene expression was categorized as upregulated/downregulated if there was an up/down 1.5-fold change in the levels within the PD diet and CD diet. Changes in expression are shown as the ratio between final and baseline values. *P < 0.05, between *TERT* groups (i.e., *TERT* Up and *TERT* Down). Dots represent outliers from the data in each intervention period for each variable analyzed. *n* = 49, both periods are considered.

We also explored the effect of changes in *TERT* expression (grouped into downregulation or upregulation) on glucose metabolism parameters. As TL maintenance is greatly dependent on *TERT* expression, here we analyzed the relationship between glucose metabolism–dependent cellular fitness and *TERT* expression. We found that those subjects upregulating *TERT* during the intervention significantly reduced their fasting plasma glucose concentrations and the degree of HOMA-IR, compared with those subjects who downregulated *TERT* (**Figure 4**).

### *TERT* and *WRAP53* expression and miRNA signature

**FIGURE 5:**
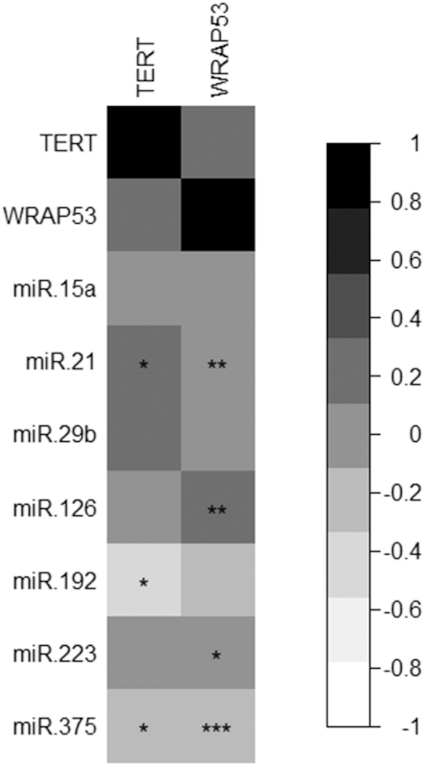
Pearson correlations between *TERT* and *WRAP53* and expression of different miRNAs related to glucose and insulin metabolism. Results represent the coefficient of correlation based on a black-to-white scale. *P < 0.05, **P < 0.01, ***P < 0.001. *n* = 49, both periods and intervention groups are considered simultaneously. miR, microRNA.

We additionally analyzed the correlations between miRNA and gene expression signature modulated after pistachio intake. Of the different Pearson correlations between *TERT* or *WRAP53* and the set of selected miRNAs, changes in miR-192 were negatively correlated with changes in *TERT* expression, and changes in miR-375 were negatively correlated with *TERT* and *WRAP*53 expression. A positive correlation was observed between miR-21 and changes in *TERT* and *WRAP53* expression. Other correlations are shown in **Figure 5**.

## Discussion

This is the first study to demonstrate a beneficial effect of pistachio intake on telomere attrition and other markers of cellular aging in prediabetic subjects. The significant upregulation in *TERT* expression and genes related to cellular aging after pistachio intake and the inverse association between telomerase expression and plasma glucose concentrations and HOMA-IR suggest a novel mechanism supporting the beneficial effect of pistachio consumption on glucose metabolism.

Several observational studies have shown a positive association between shortened telomeres, reduced telomerase activity, and T2D (23). Despite the fact that the causal role of short telomeres in the development of T2D is still unclear, experimental studies in mice deficient for the *telomerase RNA component* (*Terc*) gene have demonstrated that short telomeres might precipitate β cell senescence, giving rise to reduced β cell mass and subsequent impaired insulin secretion and glucose tolerance (24, 25). In addition, short telomeres alter the islet gene transcriptional programs affecting multiple cellular processes that are essential for insulin secretion (26). Our data support a model in which oxidative stress is increased in prediabetics not only in leucocytes but also in β cells (27) leading to telomere shortening. Short telomeres induce cellular aging–associated gene expression in β cells, which contributes to defective signaling and clinically manifests as impaired glucose homeostasis in prediabetic subjects. However, as our results were obtained with the use of only peripheral leukocytes, whether these lifestyle modifications have the same effects on β cells and adipocytes deserves further investigation. Dietary regulation of telomere attrition could therefore be a successful strategy to balance glucose metabolism and potentially decrease the risk of T2D development.

In our study we found a significant upregulation of *TERT* and *WRAP53* (telomerase Cajal body protein 1, TCAB1) expression, 2 components of the telomerase holoenzyme that play a key role in telomere maintenance (28). After pistachio consumption, we may therefore see a reduction of the rate of telomere shortening along the expected course of the subjects’ prediabetic status as the control period progresses (29). Pistachios are rich in MUFAs, genistein, resveratrol, carotenoids (lutein and zeaxanthin) (30, 31), and other phytonutrients such as anthocyans, α-tocopherol, and vitamin C, with strong antioxidant and anti-inflammatory properties. Although these phytonutrients have been associated with telomerase activation and longer telomeres (32–34), the doses of pistachio phytochemicals consumed by our participants (**Supplemental Table 4**) were relatively much lower than those that have been demonstrated to be effective in modulating gene expression and telomere activation in in vitro studies (35, 36). Hence, these phytonutrients may still regulate telomerase expression because we demonstrated that the effect of the administered amount of phytonutrients in our study was sufficient to upregulate *TERT* expression, after PD.

We can speculate that the antioxidants and various phytochemicals present in pistachios may act synergistically to modulate telomerase activation and TL. However, we cannot ignore the possibility that the effect of pistachio supplementation may also be due to changes induced by the simultaneous consumption of other food. Further investigation is needed to ascertain the potential synergistic effects of pistachio compounds.

Telomeres are highly sensitive to damage through oxidative stress due to their high guanine content (37). Oxidative damage of telomeres inhibits telomerase, leading to telomere shortening, giving rise to premature cell senescence which is involved in T2D development (38, 39). In fact, a decrease in oxidative stress will affect telomerase activation directly. Measurement of 8-OHdG in plasma therefore provides a quantitative assessment of ongoing oxidative damage or stress in the body. Telomere dysfunction induces metabolic and mitochondrial compromise, decreasing gluconeogenesis, and increasing ROS, processes related to increased IR and T2D during aging (39).

MicroRNAs, the small noncoding RNAs that regulate posttranscriptional gene expression, are significant regulators of β cell function (40). Interestingly, as described in our previous study (22), regular pistachio consumption may be an effective strategy for modulating various plasma miRNAs related to glucose metabolism and T2D (i.e., miR-21, miR-192, and miR-375). In the present study, we have shown significant correlations between *TERT* or *WRAP53* and the set of selected miRNAs modulated after PD. In fact miR-375, one of the most abundant miRNAs in the islets (41), was found to have decreased after pistachio consumption (22). This miRNA is one of the first identified in the pancreas as being able to regulate insulin secretion, having been identified as a potential circulating or tissue T2D biomarker (42). Also noteworthy is the fact that miR-375 regulates telomerase activity by reducing *TERT* transcription and activity. The exact mechanisms linking miR-375 telomerase activation with glucose metabolism are still unknown, but some evidence suggests that miR-375 is able to activate p21 and suppress telomerase activity at the transcriptional level (43). Mechanistically, our results could be rationalized by the significant decrease in circulating miR-375 after pistachio consumption being interpreted as the putative activation of TERT, improving telomere and β cell fitness and, consequently, glucose metabolism, leading to a delay in the progression from prediabetes to T2D.

The results of the present study should be interpreted in the context of its limitations. First, because this is an ancillary analysis within the framework of a crossover clinical trial, a carryover effect in TL was found in the second intervention period. For this reason the analysis of TL was conducted with the use of only data from the first period, thereby limiting the statistical power of our results. Second, the participants in our study were prediabetic, which may limit the generalizability of the findings to diabetic or healthy populations.

In conclusion, the present study supports the beneficial effects of nut consumption, and pistachios in particular, on metabolic conditions such as prediabetes, and helps to elucidate one of the potential mechanisms involved in the pathophysiology of T2D. These findings open a new line of investigation into the potential role of nuts in protecting against telomere attrition and slowing cellular aging. Whether these molecular changes could lead to a reduced risk for T2D merits further investigation.

## Supplementary Material

nqz048_Supplemental_Files

## Notes

The Western Pistachio Association (USA) and Paramount Farms supported the trial. SC was supported by a Ramon y Cajal contract (RYC-2013-12598) funded by the Spanish Ministry of Science, Innovation and Universities, with additional funding supporting this study; SG was supported by a predoctoral fellowship from AGAUR (2018FI_B_00444); JM received funding from the European Union's Horizon 2020 research and innovation program under Marie Skłodowska-Curie grant agreement 713679 and from the Universitat Rovira i Virgili (URV); JG was awarded a predoctoral grant (PFIS FI17/00255) from the Instituto de Salud Carlos III (ISCIII); and LMA received her fellowship from the “La Caixa” Foundation. The study was funded by the Spanish Ministry of Science, Innovation and Universities (RYC-2013-12598), the Navarra Government (PI 54/2009), PIUNA, and CIBERobn (project CIBER, CB06/03). CIBERobn is an initiative of the ISCIII, Spain, which is supported by FEDER funds (CB06/03). None of the funding sources played a role in the design, collection, analysis, or interpretation of the data, or in the decision to submit the manuscript for publication.

Supplemental Tables 1–4 and Supplemental Figure 1 are available from the “Supplementary data” link in the online posting of the article and from the same link in the online table of contents at https://academic.oup.com/ajcn/.

Abbreviations used: 8-OHdg, 8-hydroxydeoxyguanosine; CD, control diet; E%, energy percentage; IR, insulin resistance; LTL, leucocyte telomere length; miRNA, microRNA; ROS, reactive oxygen species; PD, pistachio-supplemented diet; T2D, type 2 diabetes; TL, telomere length.
