# Supplementary material for: Pistachio consumption modulates DNA oxidation and genes related to telomere maintenance: a crossover randomized clinical trial
Source: Am J Clin Nutr. 2019 May 3;109(6):1738–45. doi: 10.1093/ajcn/nqz048 (PMC6895461; doi:10.1093/ajcn/nqz048)

Online Supporting Material

Supplemental Figure 1. Participant flow chart of the EPIRDEM study (Consort 2010).

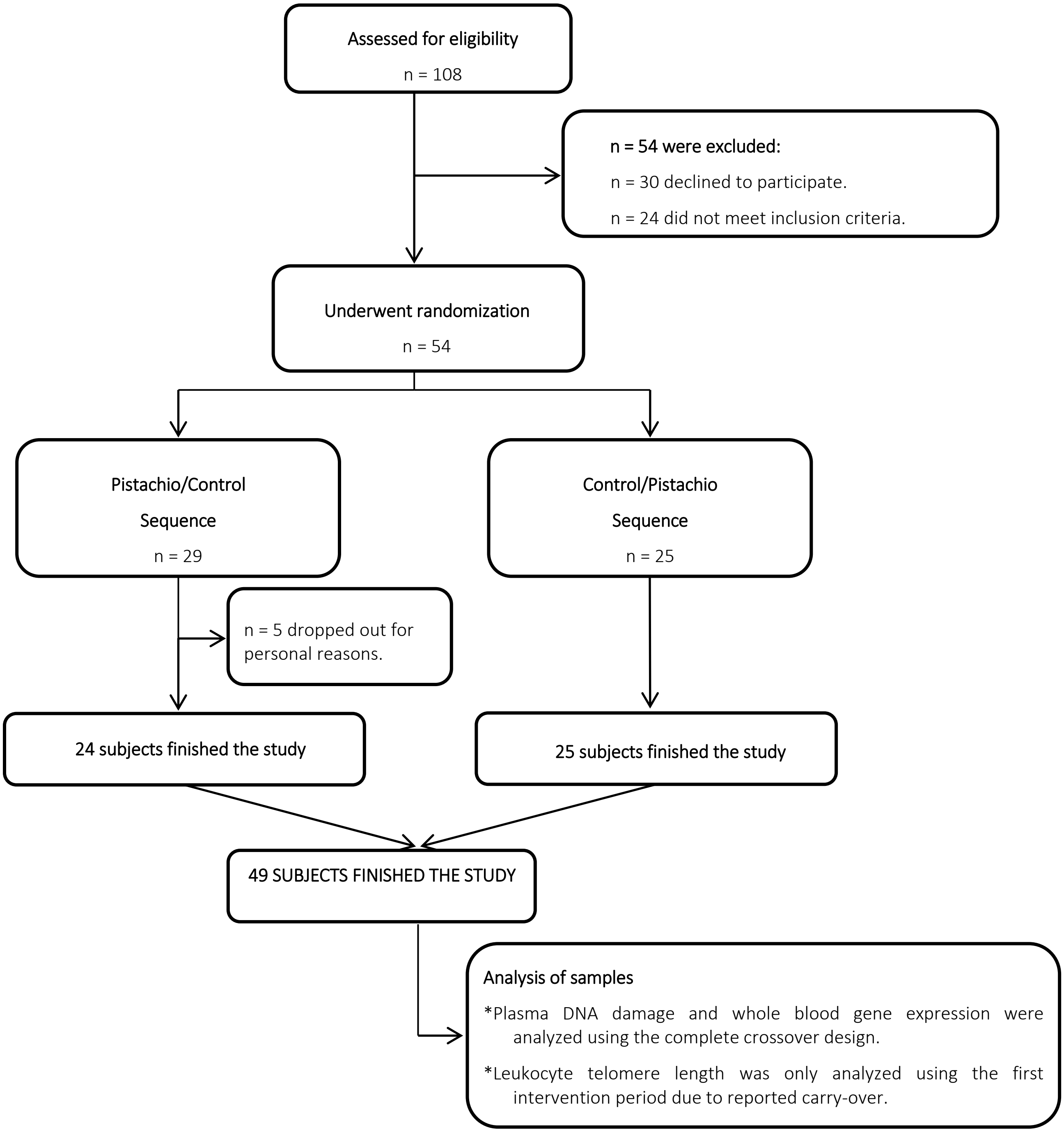

Supplement: nqz048_Supplemental_Files [file nqz048_supplemental_files.zip › OSMF1.pdf]
