# Supplementary material for: Pistachio consumption modulates DNA oxidation and genes related to telomere maintenance: a crossover randomized clinical trial
Source: Am J Clin Nutr. 2019 May 3;109(6):1738–45. doi: 10.1093/ajcn/nqz048 (PMC6895461; doi:10.1093/ajcn/nqz048)
Supplement: nqz048_Supplemental_Files [file nqz048_supplemental_files.zip › OSMT2.pdf]

Online Supporting Material

Supplemental Table 2.

Baseline DNA oxidation (ng/mL) and Telomere length (TL) as arbitrary Units (z-score adjusted). Data are given as means (95 % CI). *p* value of differences between intervention groups.

	N	Control Diet	Pistachio Diet	P value
**DNA oxidation**	49[a]	3.64 (3.37, 3.92)	3.50 (3.25, 3.76)	0.458
**TL**	24 (PD);25 (CD)[b]	0.07 (-0.16, 0.29)	-0.08 (-0.42 0.26)	0.452

Data is shown as mean (95% CI) . N as number of individuals analyzed. [a]*, both periods are considered;* [b]*, only first period is considered due to carry over effect; TL, telomere length.*
