# Supplementary material for: Pistachio consumption modulates DNA oxidation and genes related to telomere maintenance: a crossover randomized clinical trial
Source: Am J Clin Nutr. 2019 May 3;109(6):1738–45. doi: 10.1093/ajcn/nqz048 (PMC6895461; doi:10.1093/ajcn/nqz048)
Supplement: nqz048_Supplemental_Files [file nqz048_supplemental_files.zip › OSMT3.pdf]

Online Supporting Material

Supplemental Table 3. Genes considered differentially modulated by treatments in the pistachio group based on gene expression levels (Cq values <45 in PD-CD).

Gene	**Control Diet n=49**	**Pistachio Diet n=49**	**p value**
BLM	1.16 (0.99, 1.34)	1.13 (0.92, 1.33)	0.858
CHEK2	1.9 (1.2, 2.59)	1.52 (0.88, 2.17)	0.453
FOXP3	1.25 (0.87, 1.63)	1.51 (0.96, 2.06)	0.428
GPX1	1.22 (0.97, 1.47)	1.41 (1.05, 1.76)	0.308
ISG15	1.9 (0.86, 2.93)	1.74 (0.99, 2.5)	0.977
MTFP1	1.45 (0.99, 1.91)	2.1 (1.29, 2.91)	0.175
NCL	2.05 (1.41, 2.69)	1.43 (0.79, 2.07)	0.219
PPP2R1A	1.31 (0.75, 1.88)	1.27 (0.98, 1.56)	0.825
PRDX1	1.24 (0.99, 1.49)	1.46 (1.07, 1.86)	0.325
RAD1	1.28 (0.97, 1.6)	1.05 (0.9, 1.21)	0.252
RTEL1	1.4 (0.92, 1.88)	2.89 (1.31, 4.48)	0.068
SIRT2	1.26 (0.88, 1.65)	1.26 (0.83, 1.7)	0.915
SIRT6	1.15 (0.63, 1.68)	1.61 (1.04, 2.19)	0.292
SSB	1.5 (1.17, 1.83)	1.09 (0.89, 1.3)	0.072
TERT	1.01 (0.62, 1.4)	2.64 (1.33, 3.95)*	**0.043**
TINF2	1.34 (0.9, 1.78)	1.32 (0.86, 1.79)	0.928
WRAP53	0.96 (0.83, 1.1)	1.53 (1.27, 1.79)*	**0.001**

Data are given as means (95 % CI). * shows significant changes from baseline ($p < 0.05$) in each intervention period for those genes that showed significant changes between intervention periods (in bold).
