# Supplementary material for: Pistachio consumption modulates DNA oxidation and genes related to telomere maintenance: a crossover randomized clinical trial
Source: Am J Clin Nutr. 2019 May 3;109(6):1738–45. doi: 10.1093/ajcn/nqz048 (PMC6895461; doi:10.1093/ajcn/nqz048)
Supplement: nqz048_Supplemental_Files [file nqz048_supplemental_files.zip › OSMT4.pdf]

Online Supporting Material

Supplemental Table 4.

Values are expressed as IU of micronutrient per 100 g of pistachios and the daily dose (57g) of pistachio consumption in the EPIRDERM study.

Nutritional Composition of Pistachio (Dry Roasted)

Nutrient	100g	Per daily dose (57g)
**Monounsaturated fatty acids**	24.5	14
**Polyunsaturated fatty acids**	13.3	7.6
**Resveratrol (µg)**	115	65.5
**Genistein (µg)**	103.3	59
**Beta Carotene (µg)**	159	90.6
**Lutein and Zeaxanthin (µg)**	1160	661.2
**Vitamin A, IU**	266	151.6
**Vitamin B6, (mg)**	1.12	0.63
**Vitamin C, mg 3.0**	3.0	1.71
**α-Tocopherol (mg)**	2.17	1.2
**β-Tocopherol (mg)**	0.13	0.07
**γ-Tocopherol (mg)**	23.42	13.3
**δ-Tocopherol (mg)**	0.55	0.3
**Vitamin K (µg)**	13.2	7.5
**Folate (µg)**	51	29
**Choline (mg)**	71.4	40.7
**Betaine (mg)**	0.8	0.45
**Thiamine (mg)**	0.70	0.4
**Riboflavin (mg)**	0.23	0.13
**Niacin (mg)**	1.37	0.8
**Pantothenic acid (mg)**	0.51	0.3

Data obtained from the US Department of Agriculture, Nutrient Database for Standard Reference (1). Abbreviation: IU, international units.

REFERENCES

1. US Department of Agriculture, Agricultural Research Service, Nutrient Data Laboratory. USDA National Nutrient Database for Standard Reference, Release July, 2018. http://www.ars.usda.gov/ba/bhnrc/ndl. Accessed January 25
